# Role of EZH2 in cancer stem cells: from biological insight to a therapeutic target

**DOI:** 10.18632/oncotarget.16467

**Published:** 2017-03-22

**Authors:** Yiping Wen, Jing Cai, Yaya Hou, Zaiju Huang, Zehua Wang

**Affiliations:** ^1^ Department of Obstetrics and Gynecology, Union Hospital, Tongji Medical College, Huazhong University of Science and Technology, Wuhan, China

**Keywords:** EZH2, cancer stem cells, sphere, histone lysine methylation, polycomb repressive complex

## Abstract

Epigenetic modifications in cancer stem cells largely result in phenotypic and functional heterogeneity in many solid tumors. Increasing evidence indicates that enhancer of zeste homolog 2 (EZH2), the catalytic subunit of Polycomb repressor complex 2, is highly expressed in cancer stem cells of numerous malignant tumors and has a critical function in cancer stem cell expansion and maintenance. Here, we review up-to-date information regarding EZH2 expression patterns, functions, and molecular mechanisms in cancer stem cells in various malignant tumors and discuss the therapeutic potential of targeting EZH2 in tumors.

## INTRODUCTION

Tumor heterogeneity is an important feature of many solid malignant tumors and is mainly caused by a number of factors, such as the tumor microenvironment, mutations, changes in cellular hierarchy and epigenetic modifications. Although tumor heterogeneity is well explained by clonal evolution theory [1], a new hypothesis termed the cancer stem cell (CSC) model was recently proposed to account for this observed heterogeneity. This model suggests that tumors exhibit a hierarchical organization, including a small subpopulation of CSCs with stem cell-like characteristics and a large subgroup of tumor cells that have differentiated from CSCs [2]. This hypothesis is attracting much attention because it can well explain the chemo-resistance, metastasis and relapse of malignant tumors, and CSCs are an important research focus due to their potential clinical significance.

Although CSCs are widespread in many cancers, their presence still does not explain the functional and phenotypic heterogeneity observed in all tumors. Therefore, integration of the two models may better explain tumor formation [3]. For example, extrinsic environmental factors, together with chromosomal instability among the CSC population, may result in CSC phenotypic or functional heterogeneity. Many recent studies have addressed the impact of epigenetic modifications on CSC maintenance, among which the Polycomb group (PcG) genes are especially interesting within the CSC context.

PcG genes comprise a set of epigenetic regulators that catalyze specific histone posttranslational modifications. It has been reported that epigenetic modification by PcG proteins is critical for maintaining stem cell-like characteristics of adult stem cells as well as embryonic stem cells (ESCs) [4]. As a key protein in the PcG family, EZH2 mediates trimethylation of histone H3 lysine 27 (H3K27me3) and inhibition of downstream target genes [5]. More importantly, EZH2 has also been found to increase the proliferation of tumor cells and maintain the pluripotency of stem cells [4, 6]. Several studies have shown that EZH2 is aberrantly overexpressed in various malignant tumors, such as prostate cancer [7], breast cancer [8], ovarian cancer [9] and others [10, 11]. In addition, it is reported that EZH2 acts a critical factor in promoting tumor growth and metastasis in many malignant tumor models [12–14]. Therefore, as a new biomarker, EZH2 can be considered to be a novel target for the treatment of malignant tumors.

Recent studies have shown that EZH2 has a critical function in maintaining CSC properties and promoting CSC metastasis in various solid tumors as well as in leukemia. In addition, both EZH2 inhibition and knockdown (KD) dramatically decrease CSC tumorigenicity [15–19], supporting the notion that EZH2 may serve as a novel CSC marker and a potential target for cancer therapy. However, before these important discoveries can be translated into clinical applications, the specific function of EZH2 in maintaining CSC properties should be further elucidated.

## THE STRUCTURE AND FUNCTIONS OF EZH2

**Figure 1:**
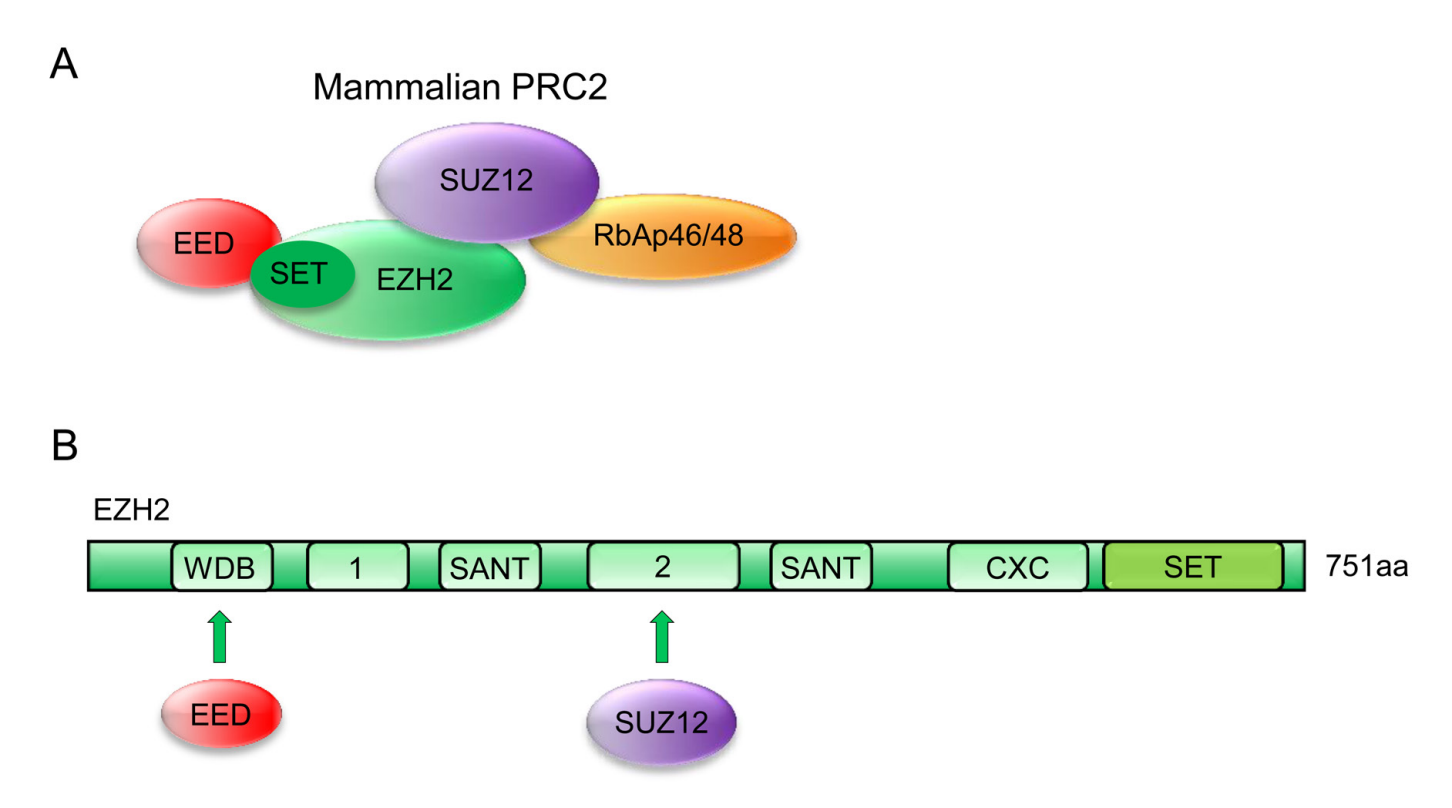
Possible architecture of the PRC2 complex **A**. Models of core human PRC2 complexes. Subunit composition and established contacts between subunits are depicted. **B**. Domain organization of human EZH2 subunits. EZH2 contains a C-terminal SET domain, an adjacent cysteine-rich CXC domain, and additional conserved regions as indicated. WDB, binding domain for EED; Domain “1”, binding region for PHF1 in human cells; domain “2”, binding region for SUZ12; CXC, cysteine-rich domain; SANT, domain that allows chromatin remodeling proteins to interact with histones; SET, catalytic domain of EZH2.

As important epigenetic regulators, PcG proteins can form chromatin-modifying complexes in a cell context-dependent manner. Two important PcG proteins, PRC1 and PRC2, have been identified in mammals [20]. The PRC1 complex includes several subunits, such as PCGF, HPH, CBX, and RING1 paralog groups, whereas the PRC2 complex mainly consists of EED, SUZ12, RbAp46/48, and either EZH1 or EZH2 (Figure 1A) [21].

Human EZH2, a 751-amino acid histone-lysine methyltransferase, is organized into several domains, among which the conserved SET domain at the C-terminus functions in maintaining histone methyl transferase (HMT) activity. Other functional domains, such as CXC and ncRBD, are required for interaction with other PRC2 components and regulatory proteins (Figure 1B) [22–24].

**Figure 2:**
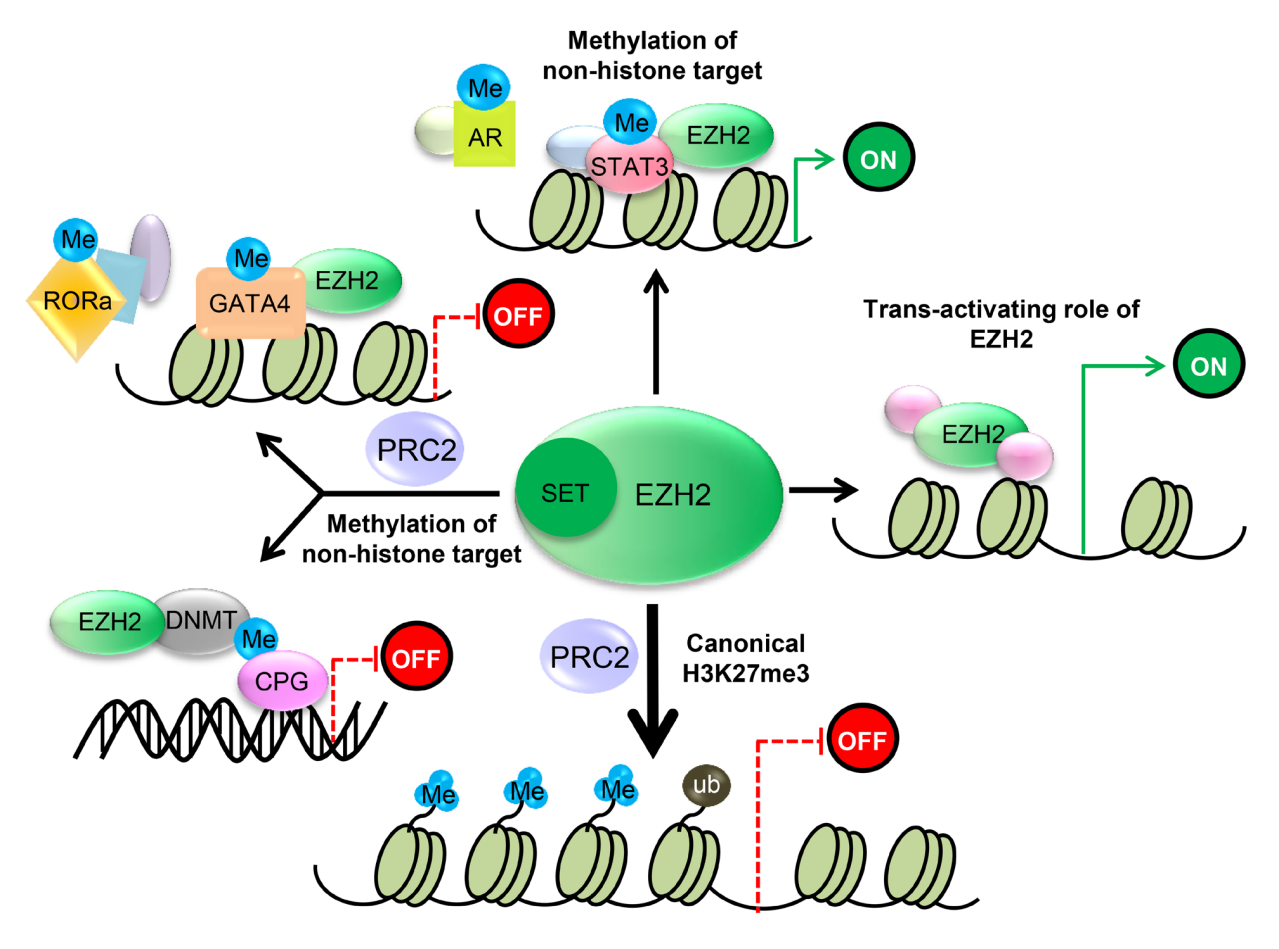
EZH2 regulates transcriptional activity 1) PRC2 methylates Histone 3 on lysine 27, which contributes to transcriptional silencing. 2) EZH2 is also capable of methylating several non-histone protein substrates, which contributes to both transcriptional silencing and transcriptional activation. 3) EZH2 also has a PRC2-independent role in transcriptional activation.

EZH2 is essential for epigenetic gene silencing. After EZH2 catalyzes trimethylation of H3K27, the PRC1 protein binds to monoubiquitinated histone H2A at lysine 119 (H2AK-119ub1) and H3K27me3. This complex then mediates chromatin compaction followed by transcriptional repression (Figure 2) [25, 26].

Recently, another mechanism for PRC2-mediated stable gene inhibition was proposed. It was reported that EZH2 directly methylates the non-histone target cardiac transcription factor GATA4 at Lys299 in a PRC2-dependent manner, thereby attenuating GATA4 acetylation by p300 and resulting in GATA4 transcriptional repression and gene silencing [27]. In addition, EZH2 was shown to bind to and methylate ROR at Lys38, followed by ubiquitination of the DDB1/DCAF1/CUL4 ubiquitin ligase complex and leading to ROR target gene silencing [28]. Moreover, EZH2 has been shown to directly bind to several DNA methyltransferases (DNMTs), such as DNMT3A, DNMT3B and DNMT1, to methylate CpG sites and mediate chromatin compaction [29] (Figure 2). These studies explored a novel mechanism in which EZH2 directly methylates non-histone targets and inhibits transcriptional activity in a PRC2-dependent manner.

In addition to transcriptional repression, emerging research shows that EZH2 has a PRC2-independent function in activating downstream genes via methylation of non-histone targets or direct binding to other proteins [30–34] (Figure 2). Previous studies showed that as a transcriptional inducer, EZH2 functions independently (not as part of the PRC2 complex) in prostate cancer, breast cancer, intestinal cancer and natural killer/T-cell lymphoma (NKTL). Xu K and colleagues demonstrated that following phosphorylation at Ser21 by AKT, EZH2 directly binds to and methylates the androgen receptor (AR), thus activating its downstream genes [31]. Another study proved that EZH2 was able to directly bind to and methylate STAT3, thereby promoting the tumorigenicity of glioblastoma and prostate CSCs [34]. Similarly, Lee ST and colleagues identified that in estrogen receptor (ER)-negative basal-like breast cancer cells, EZH2 binds to a NF-κB component to form a ternary complex, leading to transcriptional activation of its downstream target genes. These processes were independent of EZH2 HMTase activity [33]. Moreover, EZH2 has also been reported to activate target genes through the ER/Wnt/β-catenin pathway [32]. Another study by Jung HY and colleagues demonstrated that EZH2 could promote transactivation of Wnt target genes by establishing a complex with TCF/β-catenin and the DNA repair protein PCNA-associated factor (PAF), which resulted in intestinal tumorigenesis [30]. EZH2 function independent of its HMTase activity was also observed in natural killer/T-cell lymphoma (NKTL). Interestingly, EZH2 overexpression in NKTL was found not to be associated with H3K27 trimethylation [35], and ectopic expression of an EZH2 mutant lacking HMTase activity in NKTL cell lines rescued the tumor growth inhibition resulting from depletion of endogenous EZH2 [35].

In conclusion, recent findings support the hypothesis that EZH2 functions as a dual-faced molecule that acts both as a transcriptional suppressor and a transcriptional co-activator depending on its interaction with other PRC2 components and the cellular context.

## EZH2 OVEREXPRESSION IN CSCs

Compared to normal tissue cells or benign tumor cells, EZH2 is overexpressed in and promotes tumor progression in various cancers [36]. Therefore, analyses of the EZH2 expression in CSCs are of research interest.

EZH2 levels have been demonstrated to be increased in CSCs in various malignant tumors, such as melanoma cancer [17], breast cancer [37, 38], ovarian cancer [16, 39], pancreatic cancer [37], skin cancer [40], colorectal cancer [18] and leukemia [41, 42] (Table 1). Despite this evidence, the link between EZH2 and CSCs has been questioned by recent work comparing EZH2 expression in differentiated cancer cells and CSCs [43]. Surprisingly, the authors found significantly lower EZH2 expression and global levels of H3K27me3 in CD44^+^/CD24^−^ CSCs sorted from MCF7 cells and in side population cells sorted from HUH7 cells (Table 1). These findings were coupled to global DNA demethylation in CSCs compared with differentiated cancer cells. One possible explanation for this phenomenon is that CSCs comprise only a small subset of tumor cells, less than 5% even in CSC-enriched populations [44]. In addition, the CSC gene expression profile is highly dependent on the composition of the culture medium, and culturing CSCs in serum-containing media can induce CSC differentiation and abolish their self-renewal ability. A previous study also showed that the CSC differentiation status and epigenetic profile can be affected by the specific culture medium composition [45].

**Table 1:** Expression of EZH2 in CSCs

Cancer type	Cell type	CSCs	EZH2	H3k27me3	Method	References
Breast cancer	HCC193, T47D, Primary tumor	CD44+CD24-	Upregulated	Upregulated	WB; qPCR	[37]
	Primary tumor	Spheroids	Upregulated	Upregulated	WB; qPCR	[37]
	SUM149, Primary tumor	ALDH+	Upregulated	Unknown	qPCR	[38]
	MCF7	CD44+CD24-	Downregulated	Downregulated	RT-PCR	[43]
Pancreatic cancer	HPAC, Panc-1, Primary tumor	EpCam+CD44+CD24+	Upregulated	Upregulated	WB; qPCR	[37]
	Primary tumor	Spheroids	Upregulated	Upregulated	WB; qPCR	[37]
Ovarian cancer	Primary tumor	CD44+CD117+	Upregulated	Unknown	qPCR	[16]
	Ascitic fluid	Side population	Upregulated	Unknown	qPCR	[39]
	IGROV1	Side population	Upregulated	Unknown	qPCR	[39]
Melanoma cancer	WM793, A375	Spheroids	Upregulated	Upregulated	WB	[17]
Skin cancer	SCC-13	Spheroids	Upregulated	Upregulated	WB	[40]
Colorectal cancer	SW480	CD133+CD44+	Upregulated	Unknown	qPCR	[18]
	SW480	Spheroids	Upregulated	Unknown	WB	[18]
Leukemia	CML mice cells	Lin-c-Kit+Sca1+	Upregulated	Unknown	qPCR	[41]
	CML patient cells	CD34+CD38-	Upregulated	Upregulated	qPCR	[42]
Hepatocarcinoma	HUH7	Side population	Downregulated	Downregulated	RT-PCR	[43]

In general, testing the clinical significance of the CSC hypothesis requires a functional approach [46]. It is thus important to illuminate the function of a CSC-related gene within the context of a specific tumor using pharmacological inhibition and gene silencing. Therefore, in contrast to simply determining the level of EZH2 expression, identifying the functional importance of EZH2 in CSCs is of great significance and may pave the way for the clinical application of EZH2 inhibitors.

## EZH2 CONFERS CSC PROPERTIES

### EZH2 and breast CSCs

Breast cancer is one of the leading causes of cancer-associated death in women. However, the detailed mechanism remains unclear. Recent research supports that EZH2 overexpression and CSC expansion can result in the progression of breast cancer [37]. Moreover, a previous study suggested that EZH2 overexpression significantly increased mammosphere formation: each sphere overexpressing EZH2 contained two or three times more cells than the control group, which proved that EZH2 can increase the self-renewal ability of breast CSCs [15]. Similarly, a study by Gonzalez ME et al. demonstrated that EZH2 KD in primary tumor cells isolated from patients with triple-negative (TN) invasive breast carcinoma, as well as in breast cancer cell lines, significantly reduced the proportion of CD44^+^/CD24^−^ and ALDH1^+^ cells compared with controls. Moreover, reduced EZH2 expression dramatically decreased the tumorigenic ability of ALDH1+/SUM149 cells compared to control cells. However, EZH2 KD in ALDH1- populations had no significant effect on tumor onset and tumor volume [38].

### EZH2 and pancreatic CSCs

One of the most lethal malignancies, pancreatic ductal adenocarcinoma (PDAC) has a five-year survival rate of less than 5% [47]. Recent findings suggest that PDAC is characterized by overexpression of EZH2, promoting self-renewal capacity of CSCs through H3K27me3. Van Vlerken LE and colleagues [48] demonstrated that EZH2 KD significantly reduced the frequency of CSCs in PDAC. In Panc-1 cells, the EpCam+CD44+CD24+ frequency declined 1.6-fold from 12.7% in control cells to 7.8% after EZH2 loss (p < 0.001). In addition, CSC frequency among HPAC cells was similarly reduced two-fold from 13.8% under control conditions to 7% after EZH2 KD (p < 0.001). Therefore, it was concluded that EZH2 is essential for maintaining the pancreatic CSC population.

EMT and chemoresistance are important properties of CSCs [49], and a recent report suggested that EZH2 overexpression results in reduced E-cadherin levels, which may result in poor prognosis of PDAC patients [50]. It was also reported that EZH2 could increase the chemotherapy resistance of PDAC cells to gemcitabine [51].

### EZH2 and glioblastoma multiforme CSCs

Glioblastoma multiforme (GBM) is considered to be the most aggressive type of malignant glioma. Due to self-renewal and differentiation capacity, CSCs can lead to tumor initiation in GBM [52, 53]. Recently, Suvà ML and colleagues showed that pharmacologic inhibition of EZH2 in GBM spheres by 3-deazaneplanocin A (DZNep) at 5 μmol/L for 5 days resulted in a > 80% decrease in the clonogenic index. However, temozolomide treatment of GBM spheres at the same concentration as DZNep had no effect on CSC clonogenicity. In addition, these authors identified that a short *in vitro* treatment of CSCs with 5 μmol/L DZNep before transplantation dramatically improved survival of the host animal [54]. In summary, EZH2 is considered to function in maintaining the self-renewal capacity and tumorigenicity of GBM CSCs.

### EZH2 and ovarian CSCs

Epithelial ovarian cancer (EOC) is one of the most common malignancies in the female reproductive system. Similar to several other human carcinomas, EZH2 overexpression is critical for the maintenance of ovarian CSC populations. Recently, Rizzo S and colleagues reported EZH2 to be overexpressed in ovarian tumor-derived side population (SP) cells, which are stem cell-like cells enriched by chemotherapy, and demonstrated that EZH2 KD results in loss of SP cells and reduced anchorage-independent growth in ovarian tumor models [39]. This evidence suggests that EZH2 expression is increased in ovarian CSCs, which may contribute to EOC chemoresistance. Using chromatin immunoprecipitation (CHIP) and gene sequencing, Li H et al. reported 60 genes directly targeted by EZH2, with ALDH1A1 as a novel target of EZH2 [55]. ALDH1A1 has previously been reported as a CSC marker in ovarian and breast cancers [56–58], and the Li et al. study revealed that EZH2 directly increased ALDH1A1 expression in ovarian cancer cells, supporting the notion that EZH2 can increase the proportion of CSCs by promoting ALDH1A1 expression.

### EZH2 and prostate CSCs

Prostate cancer (PCa) accounts for the majority of cancer-associated deaths among men in the United States [47]. Recent studies have shown that as the most aggressive form of PCa, castration-resistant prostate cancer (CRPC) has a poor prognosis and high mortality, which has been in part attributed to the existence of CSCs. As in other cancer types, epigenetic alterations and microRNA (miRNA, miR) deregulation are considered important factors in prostate carcinogenesis [59]. The let-7 family has an important function in promoting PCa progression through CSC regulation. Kong D and colleagues found a lack of let-7 expression to be associated with EZH2 overexpression in human PCa tissues. In addition, enhanced let-7 expression led to decreased levels of EZH2 expression and inhibited the sphere-forming capacity and clonogenic ability of PCa cells. Moreover, the authors found that BioResponse 3,3’-diindolylmethane (BR-DIM) treatment increased expression of let-7 and decreased that of EZH2 in PCa cells, leading to repression of clonogenic and self-renewal capacity in these cells. In summary, these data indicate that reduced let-7 expression results in EZH2 overexpression, which may promote CSCs and contribute to PCa aggressiveness and recurrence [60]. Another study utilized immunohistochemical staining to examine the potential clinical significance of the levels of ALDH1 and EZH2 proteins in PCa. The results suggested that the expression level of ALDH1 is associated with tumor stage, lymphovascular invasion and extraprostatic extension, whereas that of EZH2 was correlated with the Gleason score and lymph node metastasis. Therefore, it was concluded that immunohistochemical analysis of CSC markers, such as ALDH1 and EZH2, can be applied as a predictor of tumor aggressiveness in PCa [61].

### EZH2 and skin CSCs

Skin cancer is one of the most common cancers in the United States, with more than 2 million people treated for nonmelanoma (basal cell or squamous cell carcinoma (SCC)) and 76,690 new melanoma cases each year [62]. Melanoma is the most aggressive type of skin cancer and has a poor prognosis [63]; the median survival time of metastatic melanoma is only 3-11 months [63–65], partly due to the chemo-resistance of CSCs to conventional therapy. As in several other cancers, EZH2 is overexpressed in the progression of benign nevi to invasive or metastatic melanoma [66, 67], and acquired functional mutations in EZH2 account for 3% of melanomas [17]. Further investigation identified that EZH2 is essential for maintaining MCS cell survival: inhibition of EZH2 with GSK126 and EPZ-6438 or EZH2 KD in WM793 and A375 cell lines reduced sphere-forming capacity as well as MCS cell invasion and migration [17].

Similarly, Adhikary G and colleagues considered SCC-13-derived spheroids to be epidermal CSCs (ECS cells) and demonstrated that EZH2 can promote the survival, invasion and tumor formation capacity of ECS cells, with associated increases in H3K27me. They also showed that inhibition of EZH2 by GSK126 and EPZ-6438 or EZH2 KD could reduce expression and activity of EZH2, resulting in decreased ECS cell sphere formation, invasion and tumorigenic capacity. Moreover, GSK126 and EPZ-6438 reduced levels of Bmi-1 and Oct4 but did not influence those of Sox2 or K15 [68]. Additionally, research in laryngeal squamous cell carcinoma showed that EZH2 overexpression in AMC-HN-8 cells could promote sphere-forming ability, chemotherapy resistance and tumorigenic ability of CSCs [69].

### EZH2 and colorectal CSCs

As one of the most common cancers, 1.23 million people worldwide are diagnosed with colorectal cancer (CRC) each year [70]. CRC stem-like cells (CCS-like cells) have recently attracted increasing attention due to their contribution to the poor prognosis of cancer patients [71, 72]. Chen JF and colleagues [18] reported EZH2 to be indispensable for CCS-like cell maintenance. These authors first identified that EZH2 KD cells exhibit significantly reduced mammosphere growth, as analyzed by sphere size (~75%), with fewer mammospheres (~50%) compared to WT or control cells. They also found the CD133+/CD44+ subpopulation of SW480 cells to decrease dramatically, from 42.8 ± 0.8% of WT cells and 43.3 ± 1.1% of control cells to 20.5 ± 0.5% of EZH2 KD cells. Additionally, they reported reduced expression of stem cell-associated genes such as Nanog and Sox2 in EZH2 KD cells and further showed that the tumors generated by EZH2 KD cells were smaller than those generated by the control group. In summary, these results demonstrate that silencing EZH2 reduces CCS-like cell properties.

### EZH2 and leukemia stem cells

The existence of CSCs, which were first reported in acute myeloid leukemia (AML) [73], is considered as a reasonable explanation for tumor initiation and propagation. EZH2 was recently identified as promoting leukemia stem cell (LSC) tumorigenesis by suppressing cell differentiation and maintaining stem cell properties [19]. It was also reported that EZH2 KD prevented initiation and maintenance of chronic myelogenous leukemia (CML) and survival of CML stem cells. Moreover, EZH2 was defined as a selective vulnerability of CML stem cells, irrespective of BCR-ABL1 mutational status [41]. Another study demonstrated that PRC2 is misregulated in CSCs in the chronic phase of CML, which is largely due to the extensive reprogramming of H3K27me3 targets in CML stem cells. In addition, treatment with either EZH2 siRNA or tyrosine kinase inhibitor (TKI) alone increased expression of H3K27me3 targets, and combined treatment enhanced these effects and decreased the proportion of CSCs in CML compared to treatment with TKI alone [42]. A study on mixed-lineage leukemia (MLL) showed that EZH2 KD or inhibition by DZNep reduced LSC frequency and repressed MLL proliferation. Further analysis showed that p16 KD could expand LSCs and inhibit prognosis improvement of DZNep-treated mice. Moreover, CHIP assays revealed that EZH2 is enriched around the transcriptional start site of the p16 gene in MLL/ENL and in Hoxa9/Meis1-transduced cells [74]. In conclusion, these data suggest that EZH2 can be regarded as a potential therapeutic target for MLL.

## MECHANISMS OF EZH2 ACTION IN REGULATING CSCS

### BMP signaling

**Figure 3:**
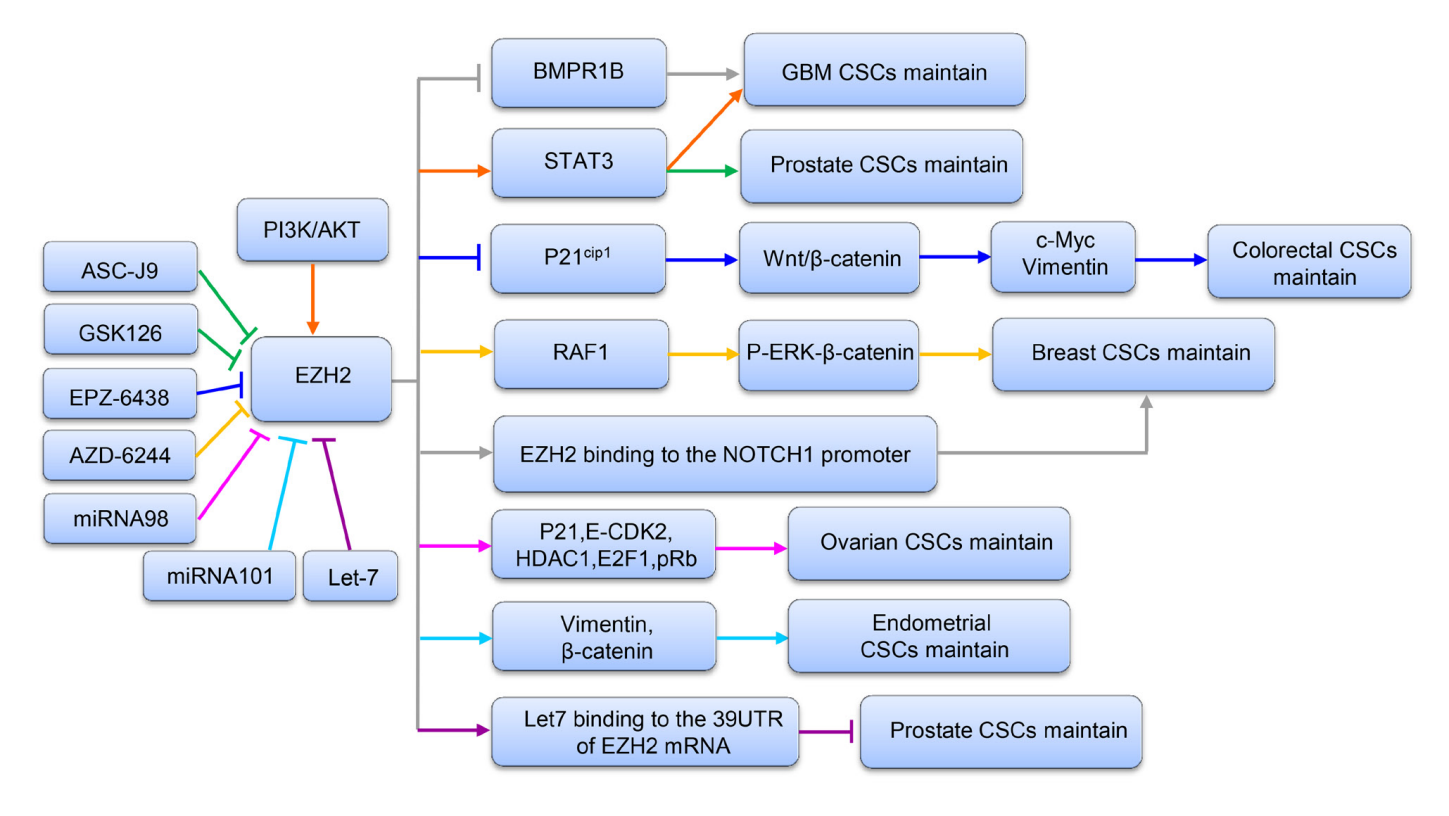
A schematic diagram illustrating the mechanism of EZH2 in maintaining CSCs in various tumors Upstream and downstream genes involved in the process are shown.

The BMP-mediated PS-SMAD1/5/8 pathway is a well-known cytokine-mediated pathway regulating normal neural stem cell (NSC) differentiation. Glioblastoma CSCs display some similarities with early embryonic NSCs; for example, they both lack BMPR1B expression and have a temporary response to BMP ligands [75]. Lee J and colleagues found that BMPR1B hypermethylation in 0308 CSCs may result in a lack of signal transduction responses to the CNTF and BMP pathways [76]. They also proved that EZH2 KD can increase expression of BMPR1B in these cells. Further investigation confirmed that EZH2 KD in 0308 CSCs dramatically decreased methylation of CpG nucleotides in the BMPR1B promoter region, indicating that deficiency in BMPR1B expression can be primarily attributed to PRC2-mediated transcriptional repression via methylation of CpG nucleotides in the BMPR1B promoter [75]. In conclusion, these data support that EZH2-mediated BMPR1B repression is essential for maintaining CSC properties in GBM, especially with regard to inhibiting differentiation (Figure 3).

### AKT/EZH2/STAT3 signaling

STAT3 signaling is regarded as a target for cancer treatment due to its important function in a variety of malignant tumors [77, 78]. Activation of STAT3 involves a series of events [79], and recent research identified that STAT3 downstream genes are overexpressed in the mesenchymal GBM subtype, supporting that STAT3 signaling has a key function in controlling mesenchymal transformation in glioma [80, 81]. Another study reported that EZH2 S21D overexpression induces STAT3 methylation and promotes STAT3 activity in GSCs compared to non-CSCs [34]. Further analysis found that inhibition of AKT signaling could mediate EZH2 phosphorylation, which in turn resulted in STAT3 inactivation. These results indicate that interactions between EZH2 and STAT3 in GSCs are regulated by upstream PI3K/AKT signaling. Therefore, it is concluded that EZH2 participates in PRC2-independent transcriptional activation by directly binding to STAT3, which results in GSC expansion and GBM progression. Similarly, Wen S and colleagues [82] indicated that EZH2-STAT3 signals influence prostate tumor progression by directly methylating the promoters of stem cell-related genes, such as Sox-2, Oct-4 and Nanog. In addition, these authors found that ASC-J9^®^, a newly identified AR degradation enhancer, could mediate inhibition of STAT3 methylation and phosphorylation in prostate CSCs by suppressing the function of EZH2. Therefore, ASC-J9^®^, as well as the EZH2 inhibitor GSK-126, may be effective at suppressing PCa metastasis by targeting EZH2-STAT3 signals (Figure 3). Together, the evidence to date suggests that inhibition of EZH2 suppresses the functions of STAT3 signals involved in CSC maintenance, supporting that EZH2 can be regarded as a target for cancer therapy.

### Wnt/β-catenin signaling

β-Catenin activation is important in maintaining the self-renewal capacity and survival of CSCs in various tumors, such as leukemia, skin cancer and gastrointestinal cancer [83–85]. As the upstream regulator of β-catenin, the wingless pathway is also reported to maintain the properties of Drosophila ovarian stem cells [86]. Moreover, in CD117^+^ ovarian CSCs, β-catenin has been identified as the gene downstream of the stem cell factor receptor [87]. The Wnt/β-catenin pathway is vital to CCS-like cell maintenance [88]. Recently, gene expression data from 433 human CRC specimens in The Cancer Genome Atlas (TCGA) database were used to analyze EZH2 expression levels relative to those of randomly chosen target genes in the Wnt/β-catenin pathway. The authors found that expression of a Wnt signature, based on combined mRNA expression of all 12 Wnt/β-catenin pathway target genes, to be positively correlated with EZH2 mRNA expression. They also found that EZH2 KD could decrease the expression levels of β-catenin and downstream target genes such as vimentin and c-Myc in SW480 and SW620 cells. In addition, β-catenin, c-Myc and vimentin levels were increased following EZH2 overexpression, with significant increases in sphere formation and the CD44^+^ population. Collectively, these findings demonstrate that EZH2 is indispensable for activating Wnt/β-catenin signaling to maintain CCS-like cell properties. In an attempt to determine the mechanism, another study showed that levels of P21^cip1^, a cell cycle regulator that can arrest the cell cycle at the G1/S phase [89], were increased following EZH2 knockdown in CD133^+^/CD44^+^ SW480 cells, whereas the levels of β-catenin, vimentin and c-Myc expression were reduced. Similarly, treatment with EPZ-6438, a specific inhibitor of EZH2 [90], increased p21^cip1^ expression and inactivated the Wnt/β-catenin pathway. These findings support the notion that EZH2 knockdown inactivates Wnt/β-catenin pathway by increasing p21^cip1^expression, resulting in G1/S-phase arrest. Similarly, EZH2 overexpression-mediated repression of DNA damage repair was found to result in RAF1 gene amplifications in breast CSCs, which promoted breast CSC expansion by activating p-ERK-β-catenin signaling. This study also revealed that AZD6244, a RAF1-ERK signaling inhibitor, could improve the prognosis of breast cancer by eliminating breast CSCs [15] (Figure 3).

### Notch signaling

Notch signaling is involved in a series of biological events, such as cell proliferation, differentiation, apoptosis and embryogenesis [91]. A previous report indicated that enhanced expression of Notch pathway components is essential for maintaining self-renewal and chemoresistance in CSCs and SP cells [92]. A recent study suggested that increased EZH2 expression could increase both NOTCH1 expression and signaling. Moreover, NOTCH1 inhibition prevented EZH2-mediated CSC expansion in breast cancer. Further study revealed that in TN breast cancer, EZH2 directly binds to the NOTCH1 promoter to active NOTCH1 signaling (Figure 3), independent of PRC2-mediated H3K27me3 [38].

### EZH2 and CSC-related genes

The epigenetic mechanism of gene repression by EZH2 is involved in CSC-associated features such as differentiation, migration, invasion and chemoresistance [15, 17, 18, 38, 39, 51, 54, 60]. The function of EZH2 in malignant tumors may due to its transcriptional repression of differentiation-related and antimetastatic genes. It has been reported that CSCs can maintain their pool by suppressing cell differentiation genes, such as p16 and p19, and that these cells exhibit other characteristics, such as decreased E-cadherin expression, that can induce metastasis [93]. A recent study identified that EZH2 can mediate p16, p19, and E-cadherin silencing through H3K27me3 [62].

c-Myc, which is critical for maintaining self-renewal and survival in glioma CSCs [94, 95], is also regarded as a key molecule involved in genetic reprogramming [96]. Suvà ML and colleagues analyzed global gene expression profile changes in primary cultures of GBM CSCs (named BT-CSCs) following treatment with DZNep for five days. As the top DZNep-deregulated gene, c-myc was chosen for further investigation. Compared with untreated cells, the clonogenic ability was decreased by 40% in c-myc-infected BT-CSCs after treatment with DZNep, an 80% decreased compared to empty vector-infected BT-CSCs. These findings suggest that c-myc partially rescues the decreased clonogenic capacity of BT-CSCs treated with DZNep. Additionally, EZH2 KD resulted in reductions in c-myc expression and depletion of c-myc transcripts by approximately 80%, further supporting that c-myc is a potential downstream target of EZH2 in the regulation of BT-CSCs [54].

### EZH2 and CSC-related microRNAs

MicroRNAs (miRNAs) are defined as short RNA molecules of 21-23 nt that block or interfere with mRNA translation to negatively regulate gene expression [97–99]. Previous studies report that specific miRNAs control CSCs in various tumors by activating or suppressing target genes [97, 99].

Liu T and colleagues showed that miRNA-98 could decrease the EZH2 expression level and inhibit the proliferation of ovarian CSCs. Moreover, EZH2 KD decreased expression of the cyclin E-CDK2 and cell cycle protein p21, as well as E2F1, HDAC1, and pRb. These authors concluded that EZH2-specific microRNA-98 could inhibit the cell cycle in ovarian CSCs by regulating the CDK-pRb-E2F pathway [16] (Figure 3).

Previous studies reported that miRNA-101 suppresses the proliferation, migration and invasion of tumor cells in various cancer types [100–104]. Konno Y and colleagues used computational analysis and microarray screening to predict three direct targets of miRNA-101, namely, FOS, EZH2 and MCL-1. Moreover, a negative correlation between the expression levels of miR-101 and its targeted genes, including FOS, EZH2 and MCL-1, was found in EC samples. In addition, knockdown of FOS, EZH2 and MCL-1 expression repressed the proliferation, migration, invasion and CSC-like phenotypes of EC cells. These results provide the first evidence that miR-101 governs multiple malignant phenotypes including proliferation, migration, invasion and cancer stemness in aggressive EC cells. This at least partly occurs through reduced EZH2 expression, followed by increased levels of p21, Bax, TIMP-3 and epithelial markers and inhibited expression of mesenchymal markers and target genes of Wnt/β-catenin signaling (Figure 3) [105].

Members of the let-7 family inhibit the progression and recurrence of several cancers by negatively regulating CSCs [106, 107]. Kong D et al. used Target Scan software to predict EZH2 as a direct target of the let-7 family. Further study proved that the let-7 family can bind to the 3’ untranslated region (UTR) of the EZH2 mRNA to decrease EZH2 expression, leading to repression of CSC self-renewal ability in prostate cancer [60] (Figure 3).

## TARGETING EZH2 FOR CANCER THERAPY

Significant experimental evidence clearly demonstrates that EZH2 participates in a series of biological events in many cancer types, including tumor initiation, progression and metastasis. Therefore, EZH2 is regarded as a potential anticancer target in current epigenetic strategies. Several EZH2 inhibitors are undergoing preclinical studies or phase 1 clinical trial, further supporting the potential clinical significance of EZH2.

### Enzymatic inhibition of EZH2

DZNep, a 3-deazaadenosine analog that can increase the expression level of S-adenosyl-l-homocysteine hydrolase (SAH) and inhibit S-adenosylmethionine (SAM)-dependent histone lysine methyltransferase activity, is a widely used EZH2 inhibitor (Table 2) [108]. Although a non-specific EZH2 inhibitor, DZNep is still able to inhibit EZH2 expression and its transcriptional repression function. The antitumor activity of DZNep in thyroid cancer is well established [109], and it is reported that DZNep significantly inhibits the sphere-forming and tumorigenic abilities of ovarian and glioblastoma CSCs [39, 54]. Nonetheless, the non-specific inhibitory effect of DZNep is a huge obstacle for its clinical application because it may also affect other SAM-dependent processes. In addition, there are remaining questions regarding its short plasma half-life and toxicity profile in animal models [110]. Therefore, it is imperative to develop DZNep analogs and select EZH2 inhibitors with increased antitumor effects and reduced toxicity. A recent study identified that the DZNep analog D9 could decrease the proliferation and tumorigenesis of CSCs, with antitumor effects in AML via inhibition of several oncogenic pathways [111]. Therefore, due to its anti-cancer effects, D9 is regarded as a potential drug candidate.

**Table 2:** EZH2 inhibitors and their status in clinical development

	Compound	Mechanism	Specificity to EZH2	Clinical Status	References
Enzymatic inhibition of EZH2	DZNep	SAH hydrolase inhibitor of methyltransferases	No	Preclinical	[108]
	D9	SAH hydrolase inhibitor of methyltransferases	No	Preclinical	[111]
	EPZ005687	SAM-competitive inhibitor of PRC2	Yes	Preclinical	[112]
	EPZ-6438	SAM-competitive inhibitor of PRC2	Yes	Phase 1/2	[90, 113]
	EPZ-011989	SAM-competitive inhibitor of PRC2	Yes	Preclinical	[114]
	El1	SAM-competitive inhibitor of PRC2	Yes	Preclinical	[115]
	GSK126	SAM-competitive inhibitor of PRC2	Yes	Phase 1	[116,117]
	GSK343	SAM-competitive inhibitor of PRC2	Yes	Preclinical	[118]
	UNC1999	SAM-competitive inhibitor of PRC2	Yes	Preclinical	[119]
Non-enzymatic inhibition of EZH2	Curcumin	MAPK pathway	No	Preclinical	[120]
	EGCG	Promotion of EZH2 proteasomal degradation	No	Preclinical	[121,122]
	SFN	Inhibitor of Ezh2 transcription	No	Preclinical	[127,128]

Recently, several potent selective inhibitors, including EPZ005687, EPZ-6438, EPZ-011989, EI1, GSK126, GSK343 and UNC1999 (Table 2), have been identified through high-throughput screening. All of these compounds act as SAM competitive inhibitors.

EPZ005687 exhibits more than 500-fold selectivity toward EZH2 compared with other methyltransferases. This compound can also inhibit H3K27me3 in various cancer cells harboring wild-type, tyrosine Y641- or alanine A677-mutant EZH2 in a dose-dependent manner [112].

Compared to EPZ005687, EPZ-6438 has a strong inhibitory effect and better oral efficacy [113]. Treatment with EPZ-6438 dose-dependently decreased H3K27me3 levels and improved prognosis in EZH2-mutant non-Hodgkin lymphoma (NHL) mouse models [90]. Currently, EPZ-6438 is being applied in phase 1/2 clinical trials of refractory B-cell lymphoma and advanced solid tumors (NCT01897571). In this study, the researchers observed partial or complete response in 9 out of 15 NHL patients, including complete response in a malignant rhabdomyoma patient and partial response in a patient with EZH2 mutation (EZH2Y646H); these results show the potential drug effects of EPZ-6438. Two other clinical trials (NCT02395601 and NCT02082977) of SMARCB1-deficient tumors and EZH2-mutant B cell NHL are underway [36]. Additionally, a recent study reported that another molecule, EPZ-011989, sharing a similar structure with EPZ-6438 could expand the inhibition range of EZH2 and improve pharmacodynamic and pharmacokinetic qualities in a mouse B cell lymphoma model [114].

El1, another SAM-competitive inhibitor of EZH2, exhibits more than 10,000-fold selectivity toward both wild-type and mutant EZH2 compared with other methyltransferases [115]. It is reported that El1 modulates H3K27me2 and H3K27me3 levels without altering EZH2 expression. El1 also exhibited an antitumor effect only in SMARCB1-mutant rhabdoid tumors and EZH2-mutant diffuse large B cell lymphoma (DLBCL) [115].

As the most potent inhibitor of EZH2, GSK126 shows greater than 1,000-fold selectivity for EZH2 compared with other methyltransferases containing SET or non-SET domains and 150-fold selectivity compared to EZH1 [116, 117]. One study showed that GSK126 was able to inhibit proliferation of EZH2 mutants in DLBCL cell lines as well as in EZH2-mutant DLBCL xenografts. Therefore, GSK126 has an important function in improving the poor prognosis of tumors with EZH2 overexpression. GSK343, as a derivative of GSK126, has been shown to exhibit antitumor activity in EOC cells [118].

UNC1999, a GSK126 analog, exhibits high selectivity for both wild-type and Y641-mutant EZH2, with good oral efficacy. Additionally, it was reported that UNC1999 reduced levels of H3K27me3 and displayed an antitumor effect in EZH2 Y641N-mutant DLBCL cells [119].

### Non-enzymatic inhibition of EZH2

In addition to enzymatic inhibition, some natural compounds have also been proven to be effective in repressing the expression level and function of EZH2 in several malignant tumor types (Table 2). It is reported that curcumin, a natural component of turmeric, can reduce EZH2 expression through the MAPK pathway rather than by increasing protein degradation, which could inhibit tumor cell proliferation [120]. Another compound, epigallocatechin-3-gallate (EGCG), is an important component of dietary omega-3 polyunsaturated fatty acids and green tea. It was reported that EGCG reduced EZH2 expression levels by increasing proteasomal degradation [121, 122]. In addition, sulforaphane (SFN), a natural tumor-preventing compound extracted from broccoli and other cruciferous vegetables [123] was shown to induce apoptosis in melanoma tumor cells and have significant therapeutic potential in skin cancer [124–126]. Previous reports identified that SFN could decrease expression of EZH2 through a proteasome-dependent mechanism [122, 127]. Recently, Tiffen J and colleagues reported that SFN reduced EZH2 expression and H3K27me3 marks in the melanoma cancer cell line A375, though these effects were not reversed by lactacystin, a proteasome inhibitor. Instead, the authors identified that SFN treatment decreased the level of EZH2 by inhibiting its transcription [128].

## CONCLUSIONS, QUESTIONS AND FUTURE DIRECTIONS

In summary, EZH2 is required to maintain CSC self-renewal capacity and tumorigenic ability and confers resistance to chemotherapy and radiotherapy. Moreover, we discuss a variety of agents targeting EZH2 that may improve cancer treatment. However, EZH2 is not always highly expressed in CSCs. For instance, the level of EZH2 is low in CSCs in hepatocarcinoma and breast cancer [43]. Thus, EZH2 expression and function should be investigated in each individual type of tumor. Moreover, there are no universal markers to date that can be used for CSC detection in all cancer types, and putative CSC markers have not been identified for every type of cancer [129]. This situation leads to ambiguity in defining CSCs and may lead to variable results regarding EZH2 expression in CSCs in different types of cancer. It important to note that low EZH2 in CSCs and the presence of compensatory regulation that overcomes EZH2 inhibition will affect the efficacy of EZH2-targeting treatments. Overall, EZH2-targeted therapy is likely to be less cytotoxic when applied as a single agent. Such treatment may be beneficial when combined with other treatments such as sensitivity enhancers in adjuvant settings.

**Figure 4:**
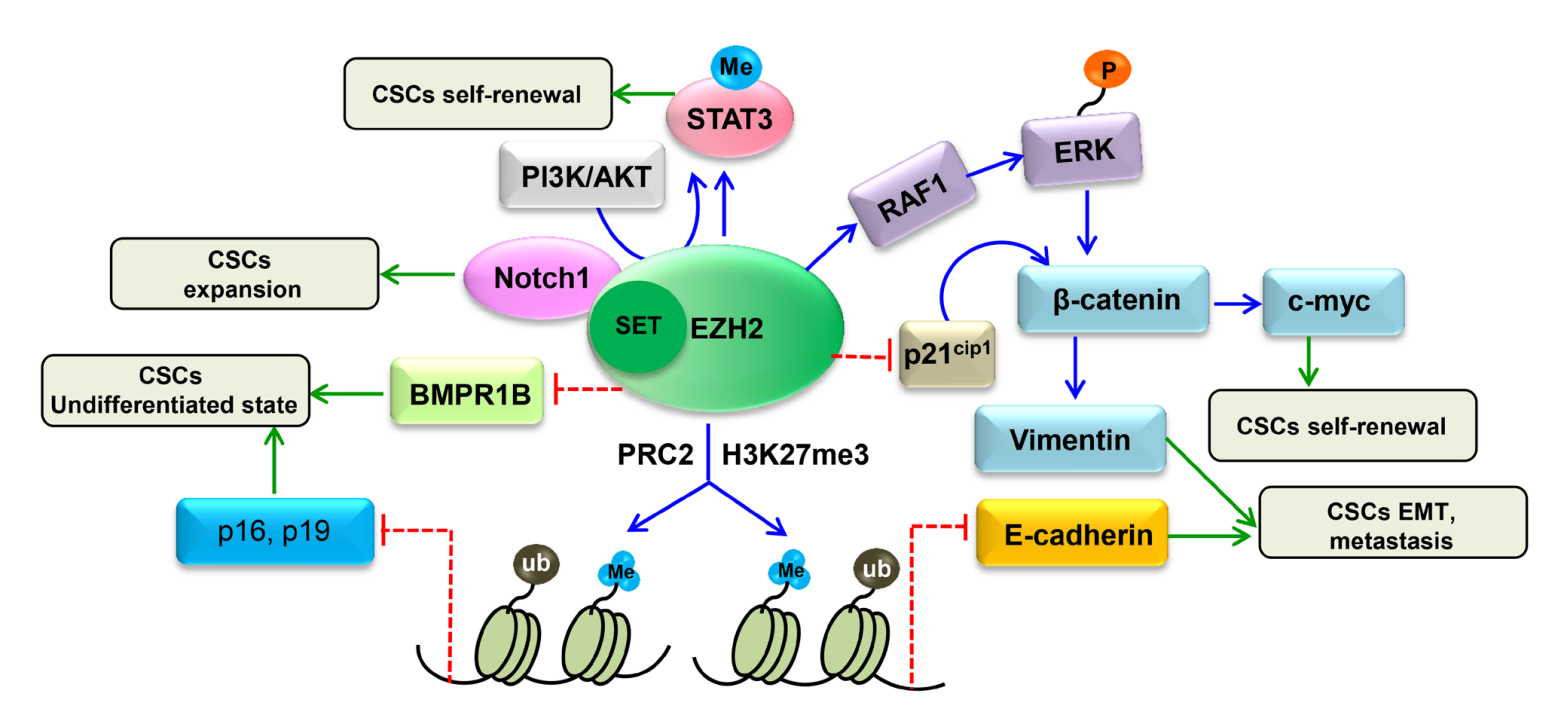
Proposed mechanism involving the regulation of CSC properties by EZH2 EZH2 regulates CSCs through multiple pathways, including BMP signaling, WNT/β-catenin signaling, AKT/EZH2/STAT3 signaling and Notch signaling. In addition, EZH2 mediates p16, p19 and E-cadherin silencing through H3K27me3 to maintain CSC properties. EZH2 also directly methylates STAT3 and inhibits BMPR1B through a PRC2-independent pathway to regulate CSCs.

In addition, interaction between EZH2 and various factors is extremely complex. The function of EZH2 occurring through PRC2 depends on the existence of other components of the complex, such as SUZ12 and EED [21]. Moreover, interplay between PRC1 and PRC2 can improve recruitment and activity of PRC2 at target sites of chromatin, which is also regulated by other types of histone modifications such as phosphorylation and acetylation, which function antagonistically with PRC2 [130]. lncRNAs, such as HOTAIR [131], Xist [132] and Meg3 [133], are also involved in controlling PRC2 function. Regarding the PRC2-independent action of EZH2, it can directly methylate non-histone targets such as GATA4, ROR and STAT3, bind to DNA methyltransferases to methylate CpGs and form transcriptional complexes with other proteins to regulate gene transcription, protein function and degradation. Although limited, emerging evidence suggests that EZH2 can regulate CSCs via both PRC2-dependent and PRC2-independent mechanisms (Figure 4). Regardless, the molecular mechanisms by which EZH2 maintains CSC properties require further investigation.

In conclusion, regardless of whether it functions as a classical oncogene or a CSC-specific regulator, novel anti-cancer therapeutic strategies will emerge from further research on the molecular mechanisms of EZH2 action in CSC regulation.

## Abbreviations

AML: acute myeloid leukemia
AR: androgen receptor
BR-DIM: 3, 39-diindolylmethane by Bio Response, Boulder, CO
CHIP: chromatin immunoprecipitation
CML: chronic myelogenous leukemia
CRC: colorectal cancer
CRPC: castration-resistant prostate cancer
CSCs: cancer stem cells
ECSs: epidermal cancer stem cells
EGCG: sepigallocatechin-3-gallate
EMT: epithelial-mesenchymal transition
EOC: epithelial ovarian cancer
EPCs: early precursor cells
ER: estrogen receptor
ESCs: embryonic stem cells
EZH2: enhancer of zeste homolog 2
GBM: glioblastoma multiforme
H3K27me3: trimethylation of histone H3 lysine 27
KD: knock down
MCSs: melanoma cancer stem cells
MLL: mixed-lineage leukemia
NHL: non-Hodgkin lymphoma
NKTL: natural killer/T-cell lymphoma
NSCs: neural stem cells
PCa: prostate cancer
PDAC: pancreatic ductal adenocarcinoma
PcG: Polycomb group
PRC2: Polycomb repressive complex 2
SAH: S-adenosyl-l-homocysteine hydrolase
SCC: squamous cell carcinoma
SFN: sulforaphane, 1-isothiocyanato-4-(methylsulfinyl) butane
SP: side population
TCGA: The Cancer Genome Atlas
TKI: tyrosine kinase inhibitor.
